# Translumbar Endovascular Coiling of an Abdominal Aortic Aneurysm Complicated by Lumbar Epidural Extravasation: A Novel Case

**DOI:** 10.7759/cureus.88262

**Published:** 2025-07-18

**Authors:** Neel H Mehta, Neil Klinger, Gabrielle Luiselli, Mohammad A Aziz-Sultan, Kurtus Dafford, Hasan Zaidi

**Affiliations:** 1 Department of Neurosurgery, Massachusetts General Hospital, Boston, USA; 2 Department of Neurosurgery, Brigham and Women's Hospital, Boston, USA

**Keywords:** abdominal aortic aneurysm, coiling, endovascular, laminectomy, neurosurgery, surgery

## Abstract

Abdominal aortic aneurysms (AAAs) require complex surgical management. Currently, endovascular aneurysm repair (EVAR), including the placement of stent grafts and endovascular coils, offers early survival benefits over open surgery but carries its own distinct set of complications. We report the case of a 68-year-old man with a 35-mm saccular AAA who initially underwent stent graft placement and later required additional endovascular treatment for a type 2 endoleak. Intraoperatively, part of the coil mass appeared to deviate from the expected contour of the aneurysm. Postoperative CT imaging revealed migration of the endovascular coils into the lumbar epidural space. To our knowledge, this is the first reported case of coil migration into the lumbar spinal canal following EVAR for an AAA. The patient developed progressive symptoms of lumbar stenosis and radiculopathy, ultimately requiring decompression, fusion, and removal of the migrated coils. This report presents a novel postoperative complication and underscores the importance of careful surgical planning, interdisciplinary collaboration among surgical subspecialists, and thorough clinical and radiographic follow-up.

## Introduction

Abdominal aortic aneurysms (AAAs), defined as an aortic diameter at least 1-1.5 times greater than normal at the level of the renal arteries [1], affect nearly 35 million adults worldwide [2]. Without treatment, AAAs may rupture spontaneously in approximately 5-7% of patients, depending on aneurysm size, leading to significant morbidity or even death [3]. Advances in surgical techniques and endovascular devices have significantly reduced the incidence of ruptured AAAs after intervention, with mortality rates for non-ruptured cases now estimated to be below 5% [4,5]. Given the potential for aneurysm growth or rupture, current management emphasizes longitudinal clinical and radiographic monitoring, with emergent repair typically indicated for unstable lesions.

Definitive treatment options for AAAs include open surgical repair using a woven graft via transabdominal or retroperitoneal approaches [6] and less invasive endovascular aneurysm repair (EVAR) techniques. EVAR involves the deployment of stent-grafts, endovascular coils, or other vascular microdevices, typically introduced through femoral artery access or, more recently, via percutaneous routes [7]. Although EVAR is clinically effective, it carries risks of device-related complications, such as endoleaks, including inadequate graft sealing at attachment sites (Type 1), retrograde filling of the aneurysm sac through branch vessels (Type 2), and leakage through defects in the graft material (Type 3) [8].

To date, neurological complications following EVAR have been rarely reported and are generally limited to ischemic sciatic neuropathy [9], spinal cord ischemia [10], or lower extremity paresthesia secondary to thromboembolism [11]. To our knowledge, there have been no documented cases of endovascular device extravasation extending beyond the aortic wall into the spinal canal.

Here, we present the case of a 68-year-old male with epidural extravasation of endovascular coils following treatment of a Type 2 endoleak in a saccular AAA. As this is a single case report, institutional review board approval was not required. The patient provided informed consent for the procedure and for publication of associated images. Due to progressive neurological symptoms, the patient underwent lumbar laminectomy and decompression (L2-L4) for removal of the migrated coils. To our knowledge, this represents the first report of extradural coil migration following AAA repair. This case highlights the importance of precise translumbar access with imaging guidance, careful consideration of the approach to endoleak repair, and diligent postoperative imaging to detect rare but serious complications.

## Case presentation

A 68-year-old male with a history of paroxysmal atrial fibrillation, sick sinus syndrome, hypertension, coronary artery disease, interstitial lung disease, and hypercholesterolemia developed worsening lower back pain in 2015. Lumbar MRI at that time revealed multilevel lumbar degenerative changes and an incidental finding of an AAA, which was further characterized by CT angiography (CTA) as a 35 mm infrarenal bilobed fusiform AAA. Longitudinal clinical monitoring was recommended.

Surveillance CTA one year later showed mild enlargement to 37 mm. Interval CTA the following year demonstrated a morphologic change from bilobed fusiform to saccular configuration, a subtype associated with increased risk of rupture [12,13]. As a result, the patient underwent an uncomplicated fenestrated EVAR (Cook ZFEN covered stent) in 2018. Postoperative and interval angiography showed satisfactory aneurysm repair, and the patient remained asymptomatic at six-month follow-up.

However, surveillance imaging a few months later revealed enlargement of the AAA with evidence of a Type II endoleak, characterized by retrograde filling of the aneurysm sac from a branch vessel in the left superior aspect of the sac. Type II endoleaks are commonly treated with translumbar, transarterial, or transcaval embolization using detachable coils delivered via a percutaneous posterior approach. In this case, progressive enlargement with persistent evidence of a Type II endoleak prompted a translumbar sac embolization via standard posterior lumbar approach, performed at an outside hospital. Intraoperative serial fluoroscopy revealed that some coils were either deployed outside or had migrated from the aneurysm sac.

**Figure 1:**
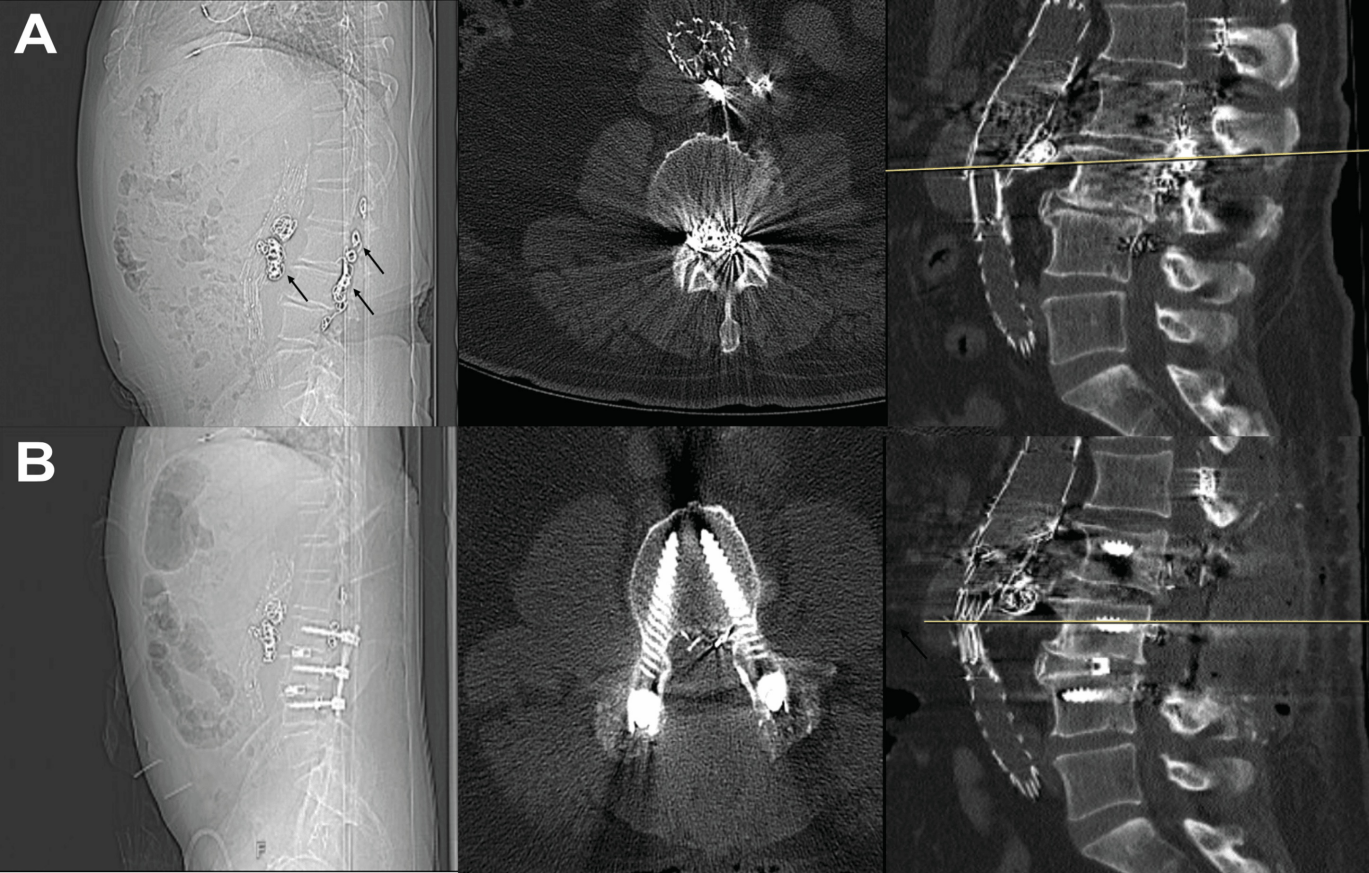
Preoperative (a) and postoperative (b) CT images of the lumbar spine Preoperative images show extravasated endovascular coils within the lumbar epidural plexus from L1 to L4 (black arrows, left panel). Stable post-interventional changes are also visible, consistent with prior fenestrated stent-grafting and secondary translumbar endovascular treatment of an AAA. On the sagittal images (right panel), yellow lines indicate the level of the axial slices shown in the middle panel for both preoperative (a) and postoperative (b) scans. AAA, abdominal aortic aneurysm

Immediately postoperatively, the patient did not report new clinical symptoms. However, CT of the abdomen and pelvis showed vascular coils within the spinal canal from L2 to L4, with possible canal narrowing at the L2 level. Two months later, CT of the lumbar spine demonstrated further coil migration into the spinal canal, with associated stenosis most severe at L2-L4 (Figure 1A, Table 1). Neurosurgery was consulted and initially recommended non-operative management with close observation.

**Table 1:** Clinical timeline from initial EVAR to lumbar decompression surgery All data were verified through manual review of the patient’s electronic medical record. Exact dates have been withheld to protect patient privacy. Key findings from representative interval imaging studies are included. AAA, abdominal aortic aneurysm; CTAP, computed tomography abdomen pelvis; EVAR, endovascular aneurysm repair; OSH, outside hospital

Date	Clinical event
January 2018	Fenestrated EVAR performed for AAA
May 2021	Interval CTAP showed a small Type II endoleak; excluded aneurysm sac measured 6.9 × 3.9 cm
May 2022	Interval CTAP revealed a persistent Type II endoleak; sac measured 7.0 × 3.8 cm
November 2022	Continued Type II endoleak on CTAP; excluded sac increased in size to 7.3 cm
February 2023	Translumbar coil embolization performed at OSH for Type II endoleak
February 2023	Postoperative CTAP showed vascular coils within the spinal canal from L2 to L4; large coil mass at L2 with possible canal narrowing
April 2023	CT of the lumbar spine showed coil migration into the spinal canal (L1-L4); moderate stenosis at L2-L3 and severe neural foraminal stenosis at L3-L4
February 2024	Neurosurgery clinic visit: patient reported significant left lower extremity weakness and intermittent pain
February 2024	Lumbar laminectomy and decompression performed for the removal of extravasated coils at L2-L4

Several months later, the patient began experiencing worsening back pain radiating to both lower extremities, accompanied by left lower extremity weakness. These symptoms were intermittent in onset and exacerbated by physical activity. Given the mass effect and suspected spinal canal stenosis caused by the intracanalicular coils, surgical intervention was pursued approximately one year after the translumbar embolization procedure.

The patient underwent L2-L4 laminectomy and complete bilateral facetectomy, followed by posterolateral fusion with transforaminal lumbar interbody fusion (Figure 1B). Some coils, such as those located dorsally at the L1 level, were not producing significant mass effect, and a preoperative decision was made to remove the majority of the coil mass localized between L2 and L4 (Video 1).

**Video 1:** Operative video of L2-L4 transforaminal lumbar interbody fusion This video demonstrates the removal of extravasated endovascular coils from the lumbar epidural plexus in a 68-year-old man.

**Figure 2:**
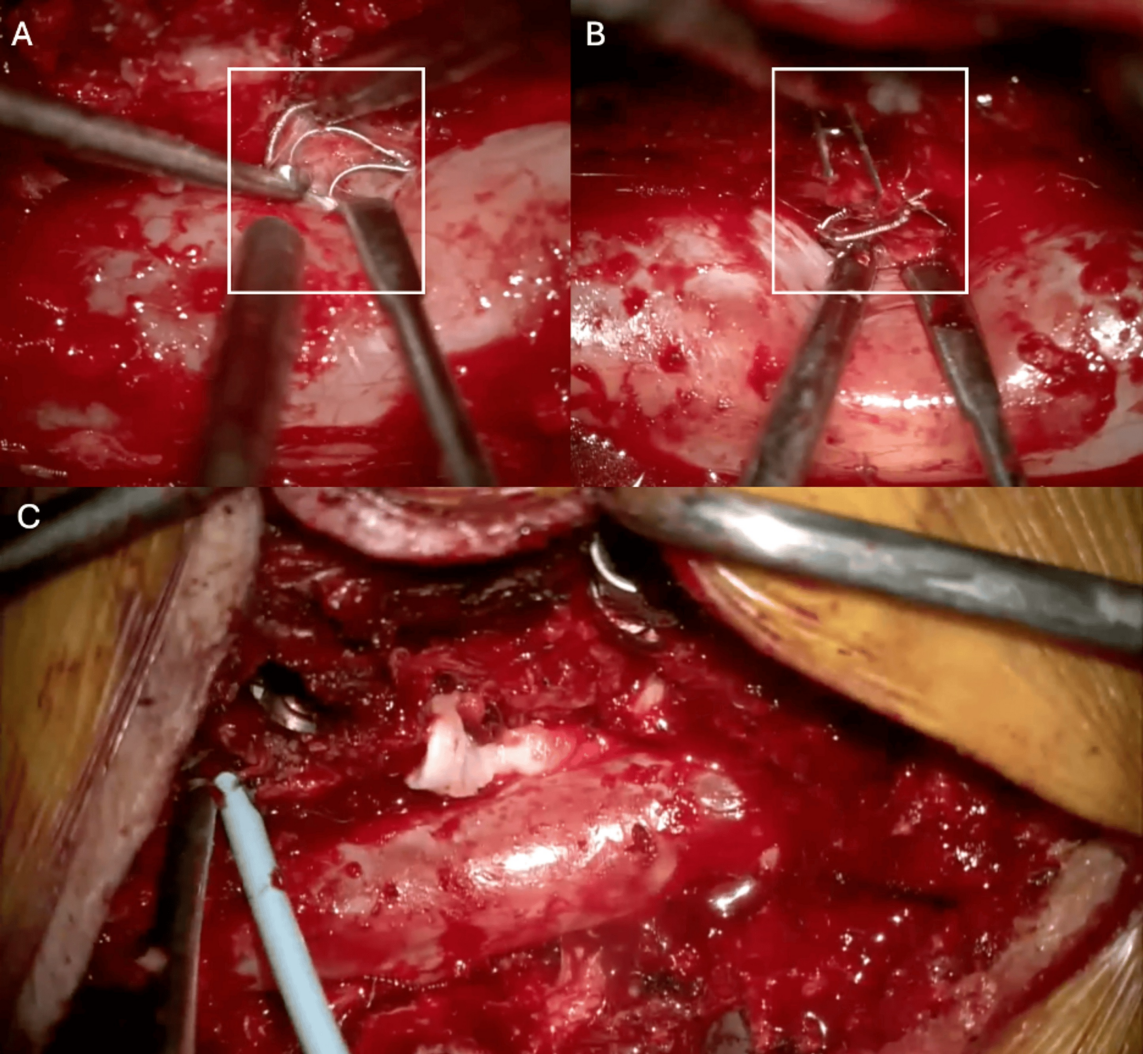
Intraoperative visualization Representative images showing intraoperative views of endovascular coils within the lumbar epidural plexus at L2-L4: (a) prior to removal, (b) during extraction, and (c) the stable postoperative surgical corridor following coil removal.

The patient was taken to the operating room and prepped and draped in standard fashion. Laminectomies and facetectomies were performed, and the ventral epidural space was explored. The coil mass was identified within epidural reactive tissue. Working bilaterally in an alternating manner, a dissection plane was developed between the coil mass and the dura. Gentle traction was applied to the free ends of the coil mass until either significant resistance was encountered or neural elements appeared to be under tension. In such cases, the coils were sharply transected. This process was repeated until the majority of the coil mass was successfully removed (Figure 2).

A substantial portion of the coil mass was found encircling the right L3 nerve dural sheath, nearly incarcerating it at the level of the L3-L4 foramen. These coils were carefully transected and peeled away from the nerve surface. The procedure was completed without complication, and no unexpected bleeding occurred. Postoperatively, the patient exhibited no new neurologic deficits and reported subjective improvement in lower extremity paresthesia. Immediate postoperative CT of the lumbar spine demonstrated adequate decompression of the coil mass with expected residual fragments (Figure 1B).

## Discussion

To our knowledge, this is the first report of extravasation of endovascular coils into the lumbar spine following translumbar endovascular coiling of an AAA. Clinical relief following lumbar decompression with coil removal underscores the need for early recognition and surgical evaluation in cases of rare intraoperative device complications.

There have been few reports of neurological complications following AAA repair and nearly no reports of device-related local effects. AAAs may result in intravascular stasis and subsequent thromboembolic disease, with transient ischemic attacks of the spinal cord during AAA repair observed in up to 1.5% of cases of thoraco-abdominal aneurysms, causing bilateral lower extremity paresthesia and subsequent paralysis [14,15]. Other reported neurologic sequelae from AAA involve spinal cord ischemic disease [16], lower extremity weakness [11], or peripheral nerve damage [17,18]. Rare complications such as paraparesis from pressure erosion of the thoracic spine have also been reported [19]. Despite these rare neurological complications, there have been, to our knowledge, no reports of extravasation of endovascular coils into the spinal epidural space.

During a translumbar approach for endoleak repair, needle catheterization is established across the spine [20]. In this setting, anatomic disruption of arterial architecture may allow for coil migration into the epidural space. At this time, it is unclear whether these coils migrated across existing vascular channels to the epidural vascular space or whether there was improper deployment during the translumbar endovascular embolization. While symptomatic lumbar stenosis should be addressed expeditiously, the appropriate timing for decompression in these cases remains unclear. These clinical nuances are further complicated by the inherent difficulty of quantifying spinal stenosis on CT imaging, with suboptimal evaluation of canal size occurring largely in the setting of metallic streak artifact from the extravasated coils.

Decompression and removal of endovascular coils is favorable prior to adherence to neural elements; however, if the coils migrate and remain intravascular, removal may create a channel between epidural vessels and the aneurysm itself, which could result in devastating vascular compromise or spinal epidural hematoma. Further, the stability of these coils within the epidural space remains to be seen, and it is unclear whether the inability to remove some coils, particularly at the L1 level, may predispose to radiculopathy, spinal stenosis, or degenerative disease later in life. While the rapid subjective improvement following decompression supports the clinical significance of coil decompression, longitudinal clinical monitoring is needed to unveil whether recurrent neurological deficits arise. Given these clinical and surgical considerations, the instinctive urge for timely intervention, especially given possible neurological compromise and clinically significant neurogenic claudication, must be balanced with careful clinical and radiographic assessment.

This novel and unexplained complication warrants additional awareness amongst the surgical community and underscores the importance of collaborative surgical care and sensitive clinical follow-up in the complex management of aneurysms [21]. This case further highlights the importance of precise translumbar access under image guidance, consideration of alternative approaches for endoleak repair, and vigilant post-procedural imaging to detect early complications. Vascular and neurosurgeons alike should remain aware of the potential for endovascular coil migration following EVAR.

## Conclusions

AAAs often necessitate complex surgical and endovascular interventions, including the use of stent-grafts and endovascular coils, which have been shown to improve clinical outcomes. Here, we report a previously undocumented case involving a 68-year-old man with a stent-grafted saccular AAA who required additional intervention for a Type II endoleak. Postoperative CT imaging revealed endovascular coils that had extravasated into the lumbar epidural space, ultimately requiring L2-L4 transforaminal lumbar interbody fusion. This case highlights a novel postoperative complication following vascular intervention for AAA and underscores the importance of precise translumbar access under image guidance, as well as regular postoperative imaging to detect rare but significant complications. Surgeons should maintain a high index of suspicion for epidural coil migration in patients presenting with new-onset radiculopathy following EVAR.
